# CHALLENGES AND SOLUTIONS FOR MOBILE HEALTH INTERVENTION RESEARCH IN OLDER ADULTS WITH MULTIPLE CHRONIC CONDITIONS

**DOI:** 10.1093/geroni/igac059.829

**Published:** 2022-12-20

**Authors:** Nada Lukkahatai, Li Junxin

**Affiliations:** Johns Hopkins University, Baltimore, Maryland, United States; Johns Hopkins University, Baltimore, Maryland, United States

## Abstract

Advances in smart phone technologies resulted in exponential growth in the use of mobile technology in health care. The mobile health or mHealth became a tool in engaging patients in self-care, monitor symptoms and provider-patients communication. Older adults living with Multiple Chronic Conditions (MCCs) are required to self-manage their complex conditions. These health demands are greater in low-income older adults. The mHealth can be beneficial in supporting health and increase access to care for this population. Our study examined a feasibility of combination of the mHealth and tailored exercise program for low SES older adults. We found that age-related physical and cognitive decline, acceptance of technology, complexity of user interface, and financial burden hinder the benefit and accessibility of the mHealth. The research team purpose solutions include improve user interface, create training session and technical support, provide incentives and technology and involve caregivers for these challenges.

